# Fasciola Hepatica Mimicking Malignancy on 18F-Fluorodeoxyglucose-Positron Emission Tomography/Computed Tomography

**DOI:** 10.4274/mirt.97759

**Published:** 2016-09-29

**Authors:** Erdem Sürücü, Yusuf Demir, Ahmet C. Dülger, Abdüssamed Batur, Şehmus Ölmez, Mehmet T. Kitapçı

**Affiliations:** 1 Yüzüncü Yıl University Faculty of Medicine, Department of Nuclear Medicine, Van, Turkey; 2 Yüzüncü Yıl University Faculty of Medicine, Department of Gastroenterology, Van, Turkey; 3 Yüzüncü Yıl University Faculty of Medicine, Department of Radiology, Van, Turkey; 4 Integra Medical Imaging Center, Ankara, Turkey

**Keywords:** Fasciola hepatica, 18F-fluorodeoxyglucose-positron emission tomography/computed tomography, Infection

## Abstract

A 48-year-old female with complaints of gastrointestinal symptoms such as abdominal pain, fatigue, vomiting, nausea, and weight loss was diagnosed with neuroendocrine tumor after removal of a 2 mm lesion from the stomach with endoscopic biopsy. Her magnetic resonance imaging that was performed due to on-going symptoms showed multiple linear hypointense lesions in the liver. Positron emission tomography/computed tomography (PET/CT) scan was performed for differential diagnosis, which showed high fluorodeoxyglucose (FDG) uptake in these lesions. Clinical and laboratory findings revealed the final diagnosis as Fasciola hepatica. The imaging features of this case is presented to aid in differentiating this infectious disease from malignancy and avoid misdiagnosis on FDG-PET/CT.

## Abstract (tr)

Kırk-sekiz yaşında karın ağrısı, yorgunluk, bulantı, kusma ve kilo kaybı şikayetleri ile başvuran kadın hastada midede nöroendokrin tümör saptandı. Şikayetlerinin devam etmesi üzerine yapılan manyetik rezonans görüntülemede karaciğerde multipl odakta lineer hipointens alanlar saptandı. Ayırıcı tanı amacıyla pozitron emisyon tomografisi/bilgisayarlı tomografi (PET/BT) çekimi için kliniğimize refere edilen hastanın PET/BT görüntülerinde multipl odakta yüksek florodeoksiglukoz tutulumu gözlendi. Hastaya klinik ve laboratuvar tetkikleri ile Fasciola hepatica tanısı kondu. Bu olgu, enfeksiyöz hastalıkların maligniteden ayrımı ve PET/BT’de yanlış değerlendirmenin önlenmesi amacıyla bildirilmektedir.

## INTRODUCTION

asciola hepatica is a helminth that causes the liver fluke disease called fascioliasis. The disease can lead to liver cirrhosis, liver failure and biliary tract obstruction (1). Fascioliasis is mostly seen in England and Ireland in Europe, northern Iran, northern Africa, Egypt, Cuba, especially the Peruvian and Bolivian Andes in South America (2).

^18^F-fluorodeoxyglucose-positron emission tomography/computed tomography (FDG-PET/CT) is a frequently used imaging tool for the management of oncologic patient and has lead to significant changes (3). In recent years, applications of ^18^F-FDG-PET/CT have become popular in non-oncological conditions such as infection and inflammation, central nervous system disorders, and cardiovascular disease (4,5,6). It can be used both for diagnosis and evaluation of therapy response.

Active granulomatous and infectious disease such as tuberculosis and sarcoidosis, infection or recent instrumentation can cause high FDG uptake in involved areas and lead to confounding results (7,8,9). So, the differential diagnosis of malignancy and infection-inflammations in PET/CT is very important for patient management and for avoiding unnecessary invasive procedures. Knowledge on the laboratory findings, clinical and imaging signs of infectious diseases can preclude the interpreter from misdiagnosis, and the patients can be evaluated more effectively. In this case, we aimed to report the PET/CT images of Fasciola hepatica in a patient with neuroendocrine gastric canceri.

## CASE REPORT

**Figure 1:**
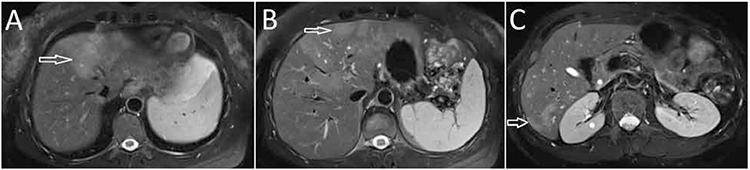
A) Axial T2W magnetic resonance image showing extensive hyper-intense lesions along the portal triads on the left lobe of the liver (arrows), B) A contrast-enhanced T1W magnetic resonance image showing multiple, round, clustered hypo-intense lesions with peripheral contrast enhancement (arrows) in the liver, C) Axial T2W magnetic resonance image showing residual parenchymal hyper-intensity after treatment (arrows)

**Figure 2:**
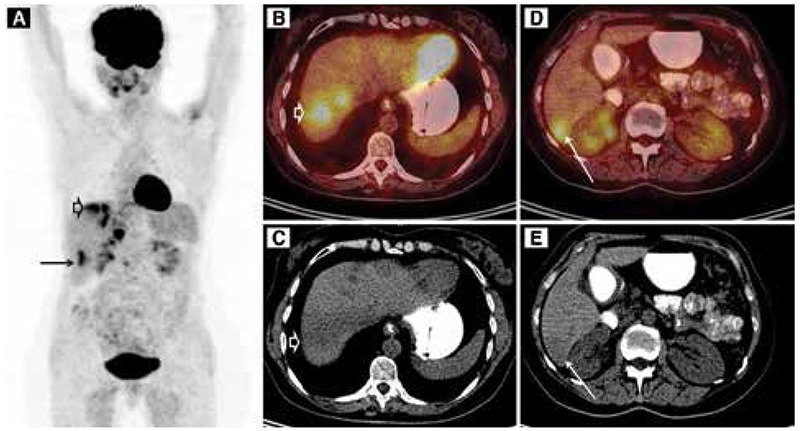
Positron emission tomography/computed tomography (PET/CT) findings of Fasciola hepatica. PET/CT scan was performed 60 min after i.v. injection of 256 MBq of ^18^F-fluorodeoxyglucose (FDG), using a lutetium oxyorthosilicate crystal equipped PET/CT (mCT20 Siemens, Germany). Maximum intensity projection images (A) show high FDG uptakes in different areas of the liver. Axial CT (C, E) images demonstrate hypodense lesions. FDG uptake (SUV_max_: 5.8) in the 7^th^ (arrow heads) and (SUV_max_: 5.2) 6^th^ liver segments (arrows) on axial PET/CT fusion images (B, D)

A 48-year-old female with complaints of gastrointestinal symptoms such as abdominal pain, fatigue, vomiting, nausea, and weight loss was admitted to the gastroenterology clinic in order to perform an endoscopy. The endoscopy revealed multiple polypoid lesions with a diameter of 1-2 mm. The histopathology examination showed grade 1 neuroendocrine tumor and incomplete metaplasia. After polypectomy, since the symptoms of the patient did not relieve completely, she was referred to magnetic resonance imaging (MRI) for detection of possible metastasis or any other etiology. MRI showed multiple linear hypointense lesions in the liver (Figure 1). A PET/CT scan was performed for differential diagnosis, which showed high FDG uptake in these lesions (Figure 2). However, the findings in FDG-PET/CT were not thought to be related to metastasis since it is known that grade 1 neuroendocrine tumor does not have FDG avidity. Thus, this uptake was thought to be associated with either a primary liver malignancy or infectious process. The patient was evaluated in detail with clinical and laboratory analysis. Indirect hemagglutination test for Fasciola hepatica was positive at 1/320. The eosinophil count has elevated to 3x10^3^ (normal range: 0-0.4x10^3^) and the EO% was 34.6% (normal range: 0-5%). All other laboratory test results were within normal range. Laboratory analysis indicated Fasciola hepatica. The imaging features of this case is presented to aid in differentiating this infectious disease from malignancy and avoid misdiagnosis on FDG-PET/CT.

## DISCUSSION

The gold standard for the diagnosis of Fasciola hepatica is serologic studies. In this case, final diagnosis was confirmed with serologic findings.

There are no pathognomonic imaging findings for fascioliasis. Ultrasound cannot detect fascioliasis. In the acute stage of the disease, CT is the golden standard for imaging. Iron oxide enhanced MR imaging may also be used (10). On CT, the disease usually appears as multiple, small, round, oval, hypodense hepatic lesions with peripheral contrast uptake. On T2-weighted MRI, a capsular hyperintensity is detected in the area where the parasite has penetrated (11). It has been reported that fascioliasis may simulate cholangiocarcinoma on MRI (12).

Firstly, it has been thought that the FDG uptake was related to a malignancy (i.e. hepatocellular cancer?). In this case, the clinician could not decide on the nature of the liver disease (whether metastatic or not) based on the MRI. Thus, he wanted to evaluate the liver further with PET/CT. However, when all the patient’s findings were evaluated together, malignancy was excluded based on the final serologic finding.

These misinterpretations have significant impact on patient management. That’s why lesions mimicking malignancy, especially on imaging modalities, must be well-known. Clinical suspicion for fascioliasis should be raised in patients who present with non-specific symptoms in endemic areas (13). Imaging modalities prevent patients from unnecessary invasive diagnostic procedures. The interpretation of artifacts, benign causes, and physiologic variants of 18F-FDG imaging is important for tumor staging. It has been reported that several entities mimic malignancy on PET/CT (7,14,15). Infectious diseases can also be evaluated with PET/CT, with a high clinical impact by providing additional diagnostic information (16). A few cases with mild FDG uptake in small Fasciola lesions have been reported in the literature (10,11). In this case, fascioliasis was detected in large areas. To the best of our knowledge, this is the first case in the literature showing extensive fascioliasis of the liver. Since FDG-PET/CT evaluates metabolic characteristics of the lesions, it also helps to detect treatment response. Currently, serial serologic testing is being used for evaluating treatment response in our patient along with radiologic evidence. The metabolic characteristics of the lesions on PET/CT can be more helpful in deciding response to therapy in comparison to CT and MRI.

## Ethics

Informed Consent: Consent form was filled out by all participants.

Peer-review: Externally peer-reviewed.

Financial Disclosure: The authors declared that this study has received no financial support.
